# Inflammation across the spectrum of hypertrophic cardiac phenotypes

**DOI:** 10.1007/s10741-023-10307-4

**Published:** 2023-04-28

**Authors:** Rosa Lillo, Francesca Graziani, Francesco Franceschi, Giulia Iannaccone, Massimo Massetti, Iacopo Olivotto, Filippo Crea, Giovanna Liuzzo

**Affiliations:** 1 Department of Cardiovascular Sciences, Fondazione Policlinico Universitario A. Gemelli IRCCS, Largo A. Gemelli 8, Rome, 00168 Italy; 2 Department of Cardiovascular and Pulmonary Sciences, Catholic University of the Sacred Heart, Rome, Italy; 3 Department of Emergency Medicine, Fondazione Policlinico Universitario A. Gemelli IRCCS, Catholic University of the Sacred Heart, Rome, Italy; 4 Cardiology Unit, Meyer Children’s Hospital IRCCS, Florence, Italy

**Keywords:** Inflammation, Cardiomyopathy, Hypertrophy, Fabry disease, Hypertrophic cardiomyopathy, Cardiac amyloidosis

## Abstract

The hypertrophic cardiomyopathy phenotype encompasses a heterogeneous spectrum of genetic and acquired diseases characterized by the presence of left ventricular hypertrophy in the absence of abnormal cardiac loading conditions. This “umbrella diagnosis” includes the “classic” hypertrophic cardiomyopathy (HCM), due to sarcomere protein gene mutations, and its phenocopies caused by intra‐ or extracellular deposits, such as Fabry disease (FD) and cardiac amyloidosis (CA). All these conditions share a wide phenotypic variability which results from the combination of genetic and environmental factors and whose pathogenic mediators are poorly understood so far. Accumulating evidence suggests that inflammation plays a critical role in a broad spectrum of cardiovascular conditions, including cardiomyopathies. Indeed, inflammation can trigger molecular pathways which contribute to cardiomyocyte hypertrophy and dysfunction, extracellular matrix accumulation, and microvascular dysfunction. Growing evidence suggests that systemic inflammation is a possible key pathophysiologic process potentially involved in the pathogenesis of cardiac disease progression, influencing the severity of the phenotype and clinical outcome, including heart failure. In this review, we summarize current knowledge regarding the prevalence, clinical significance, and potential therapeutic implications of inflammation in HCM and two of its most important phenocopies, FD and CA.

## Introduction

**Fig. 1:**
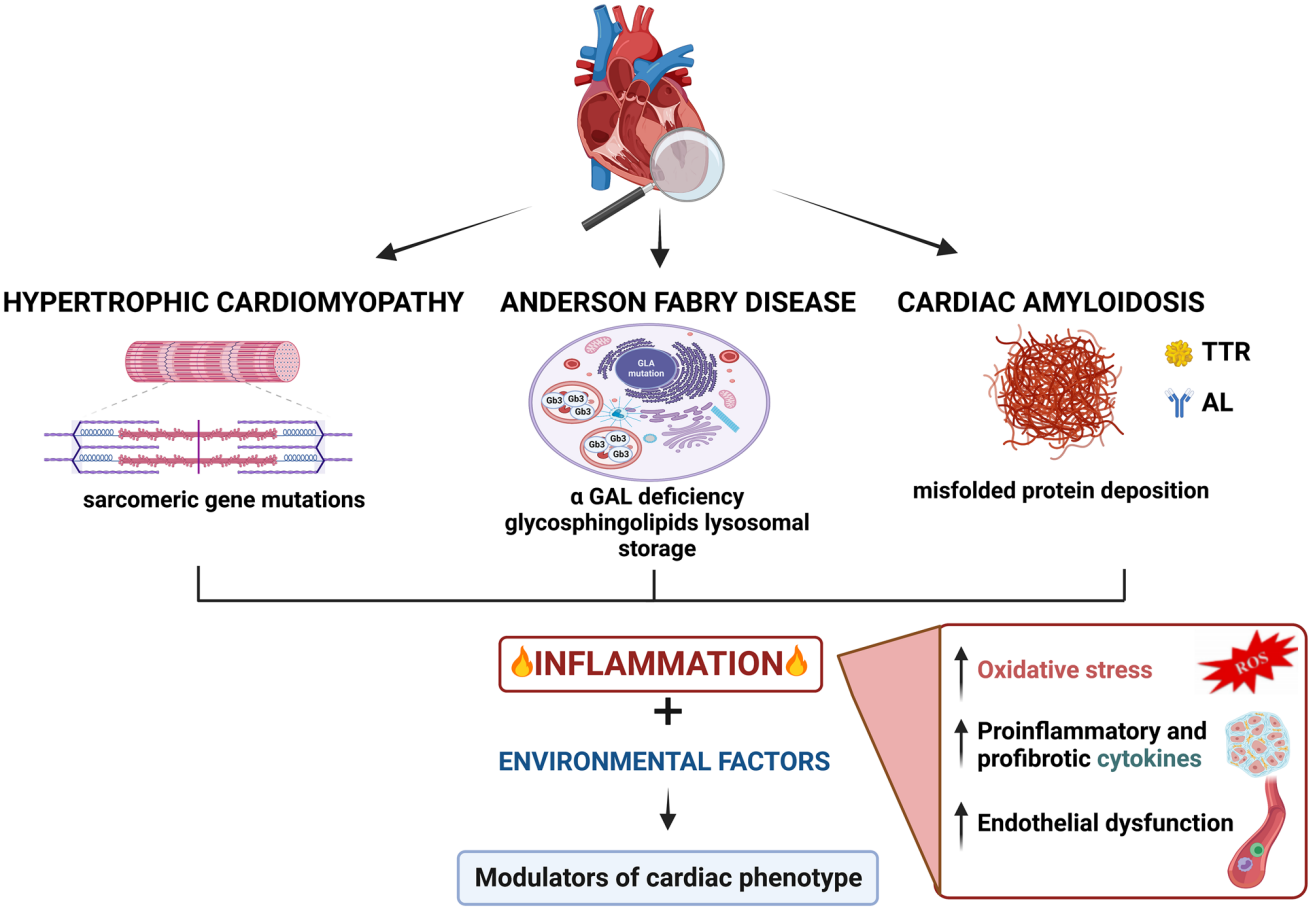
Inflammation as potential modulator of cardiac phenotype in hypertrophic cardiomyopathy, Fabry disease, and cardiac amyloidosis (created with *BioRender*)

The spectrum of cardiomyopathies with hypertrophic phenotype encompasses heterogeneous diseases, including classic hypertrophic cardiomyopathy (HCM), due to sarcomere protein gene mutations and several diseases mimicking HCM, the so-called phenocopies [1]. These conditions are characterized by different etiology, heterogeneity in penetrance, and a broad phenotypic variability, even among patients with the same pathogenetic mutation [2–4]. Thus, the final morpho-functional and clinical profiles result from a complex interaction between genotype, cellular signaling pathways, and environmental stressors (Fig. 1).

In this scenario, systemic inflammation recently gained attention as a possible key pathophysiologic process potentially involved in the pathogenesis of disease progression, ultimately influencing the severity of the cardiac phenotype and clinical outcome, including heart failure (HF) [5]. Inflammation-induced oxidative stress, mitochondrial dysfunction, impaired calcium handling, and lipotoxicity are all mechanisms that can contribute to cardiomyocyte hypertrophy and dysfunction, extracellular matrix accumulation, and microvascular dysfunction [6]. Cardiac hypertrophy due to sarcomere HCM, Fabry disease, and amyloidosis are all very different processes with fundamentally different molecular causes. Cardiac hypertrophy “per se” is not associated to inflammation, but current evidence suggests that it may contribute to define the phenotypic and clinical profile of HCM and phenocopies [5–9] even if currently it is not clear how these are connected in terms of inflammation. Yet, this aspect has been little appreciated in clinical practice despite potential therapeutic implications. The aim of this review is to summarize current knowledge regarding the prevalence, clinical relevance, and potential therapeutic implications of inflammation in HCM and two of its most relevant phenocopies, Fabry disease (FD) and cardiac amyloidosis (CA).

## Hypertrophic cardiomyopathy

### Pathophysiology: potential role of inflammation

HCM is the most common genetic cardiac disease, with a prevalence of 1:500 [10]. It is caused by mutations in sarcomere genes, coding for proteins involved in the cardiomyocyte contractile apparatus [2]. To date, the mechanisms by which sarcomere gene mutations cause myocardial hypertrophy are not fully understood, but knowledge about this topic grew during the last decades and some hypotheses have been proposed [11]. An extensive body of literature strongly supports a direct impact of sarcomere gene variants on cardiac contractility as the central cause of HCM; mutations can be associated with enhanced late sodium current activation, cellular calcium overload, and increased calcium sensitivity of the myofilaments, causing increased contractility and affecting myocardial relaxation and diastolic function [11–15]. Excessive energy consumption, in turn, causes structural and functional impairments of the mitochondria, leading to increased production of reactive oxygen species (ROS) and resulting in glutathione acylation of muscle filaments [16–20]. Moreover, impaired autophagy [18] and accumulation of metabolic end-products [20] may exert a toxic effect on the myocardial contractile apparatus and the cardiomyocyte in general. The link between sarcomere gene mutation and inflammation is not fully explained yet, but some authors [5] have supposed that cardiomyocyte disarray, sarcomere injury, mitochondrial oxidative stress, and microvascular disease may all trigger early inflammation in HCM, and some molecular mediators have been identified. Specifically, oxidative stress and inflammation modulate signaling pathways that are crucial for cardiac function, namely, AKT, ERK1/2, c-Jun, and NO-sGC-cGMP pathways. ROS induce endothelial dysfunction by attenuation of NO-sGC-cGMP signaling and contribute to an increased titin-based myocardial stiffness. Furthermore, ROS modulate post-translational modifications of other proteins involved in excitation–contraction coupling such as troponin I, cardiac myosin, and myosin-binding protein C, representing important contributors of the impaired mechanical properties observed in HCM [21]. Among molecular mediators, TNF-α cardiac overexpression is implied in LV hypertrophy (LVH), and, in turn, it induces the expression of pro-inflammatory molecules and interleukins such as IL-6 [22]. The latter is a molecular mediator of LV hypertrophy, myocardial fibrosis, and LV dysfunction in response to pressure overload. Moreover, a potential pathogenetic role of neutrophil extracellular traps (NETs) has been proposed [23]. In response to pro-inflammatory agents and ROS, neutrophils release their nuclear material, forming a web-like extracellular network. These webs, formed by DNA, histones, and neutrophil granule constituents, are named NETs. NETs represent part of a continuum of sterile inflammation and thrombosis, and they may trigger microvascular dysfunction and thrombosis, contributing to tissue injury and perpetuating cycles of ischemia and reperfusion [24], inflammation, fibrosis, and ventricular remodeling [25]. However, microvascular ischemia is multifactorial in HCM, and all stages of the ischemic process have been described at post-mortem [26]. Recurrent ischemic events themselves are likely contributors to ROS generation, myocardial inflammation, and edema, suggesting a “vicious circle” between inflammation and coronary microvascular dysfunction (CMD).

### Evidence of low-grade chronic inflammation

Several studies provided evidence of a “chronic low-grade” inflammatory state in HCM, characterized by increased levels of inflammatory cytokines, such as the aforementioned TNF-α, high-sensitivity C-reactive protein (hs-CRP), and inflammatory interleukins (i.e., IL-1β, IL-1RA, IL-6, IL-10, circulating monocyte chemoattractant protein 1) [7]. Histological studies support the presence of mild chronic inflammatory cell infiltration [27, 28], found in up to 48% of myocardial samples from patients undergoing septal myectomy [27]. Recently, Yuichi J. Shimada et al. [29] performed a large-scale investigation with comprehensive proteomics profiling in HCM, showing that Ras-MAPK (mitogen-activated protein kinase) pathway and TGF (transforming growth factor)-β, along with their upstream and downstream pathways, are selectively upregulated in HCM compared to hypertensive controls with secondary LVH.

### Emerging role of inflammation in phenotypic expression, severity, and prognosis

**Fig. 2:**
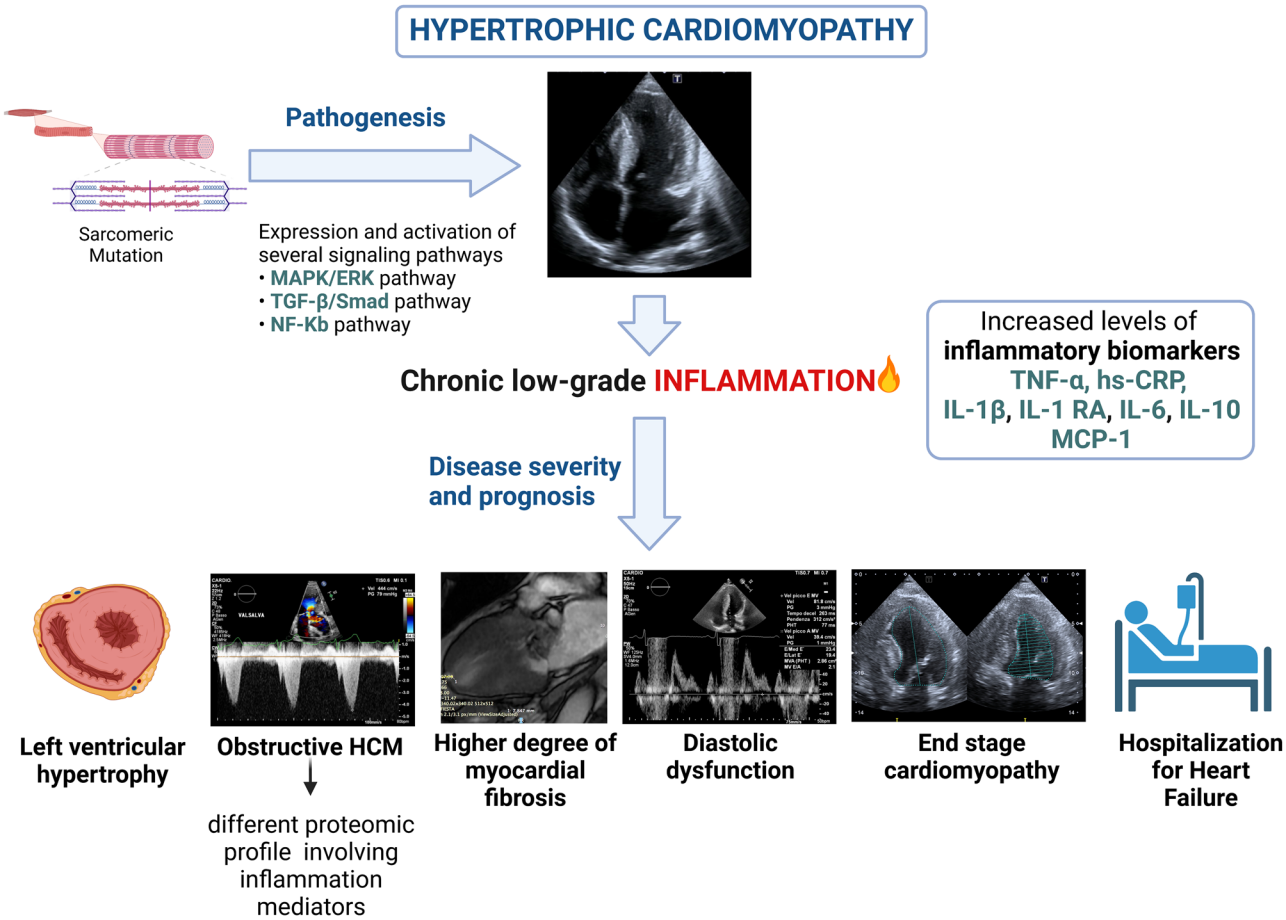
Role of inflammation in hypertrophic cardiomyopathy (HCM). In HCM several intracellular signaling pathways involving also inflammatory mediators are upregulated and a “chronic low-grade” inflammatory state has been documented. Inflammation likely plays a role in the complex interplay between genotype and phenotype, influencing disease severity (cardiac phenotype, degree of hypertrophy, myocardial fibrosis, diastolic dysfunction) and prognosis, including heart failure (created with *BioRender*)

In HCM, systemic inflammation is associated with degree of hypertrophy, myocardial fibrosis, and LV diastolic dysfunction, suggesting that inflammatory markers may be introduced to assess disease severity [30] (Fig. 2). Specifically, plasma proteomics suggests that the left ventricular outflow tract obstruction is associated with a different proteomic profile involving inflammation mediators and that surgical myectomy results in a reduction of circulating plasma proteins associated with a proinflammatory state in obstructive patients [31].

Consistent evidence exists of the pathogenetic role of inflammation in the phenotypic expression of myocardial fibrosis in HCM patients. Kuusisto et al. [7] *histologically* confirmed the presence of low-grade inflammation in the myocardium of HCM patients. Endomyocardial samples showed variable degrees of inflammatory cell infiltration and nuclear factor kappa B (NF-kB) activation. A significant association between the degree of myocardial inflammatory cell infiltration, hs-CRP, and fibrosis was found, both in histopathological samples and as myocardial late gadolinium enhancement (LGE) at cardiac magnetic resonance (CMR). The authors proposed that myocardial fibrosis in HCM is likely to be an active process, in which primary injury (caused by mechanical stress, disorganized sarcomeric and cellular architecture, microvascular ischemia, etc.) induces NF-kB upregulation in the myocardium. NF-kB, in turn, activates inflammatory cell invasion into the myocardium, the production of proinflammatory cytokines, and the fibroblast activation, leading to fibrosis. In a study by Pelliccia et al. [32], high levels of NF-kB at baseline proved predictive of worsening HF in asymptomatic/mildly symptomatic HCM during a 10-year follow-up, suggesting a role for NF-kB titration in risk stratification. Furthermore, it has been proposed that the Fas/Fas-ligand (Fas-L) system and proinflammatory cytokines may play a role in the progression to HCM end-stage phase. Zen et al. [33] found that soluble Fas (sFAS), TNF-α, and IL-6 were significantly increased in end-stage HCM, although only IL-6 was significantly different when compared with non-dilated HCM. In this latter condition, TNF-α was negatively correlated with fractional shortening, while in the dilated phase, high sFAS levels were associated with higher incidence of worsening HF. Recent studies on HCM mutant mice demonstrated that the transition from HCM to a dilated phenotype involves proinflammatory and profibrotic signaling, suggesting that therapies directed at tissue-specific inflammation and NETs may be a novel and impactful strategy for HCM [34].

Another study supporting the potential prognostic role of inflammation in HCM found an association between upregulation of the Ras-MAPK and inflammation-related pathways and occurrence of cardiovascular events [29]. The prognostic role of inflammation was confirmed by Ozyilmaz et al. [35], who demonstrated that neutrophil-to-lymphocyte ratio (NLR), a marker of oxidative stress damage, was significantly higher in patients with HCM compared to a control group and that a high NLR was associated with increased 5-year risk of sudden cardiac death in HCM patients.

## Fabry disease

### Pathophysiology: potential role of inflammation

Anderson-Fabry disease (FD) is a rare (OMIM #301500) X-linked lysosomal storage disorder characterized by intracellular accumulation of neutral glycosphingolipids (Gb3) as a result of genetic enzyme α-galactosidase A deficiency [36]. FD is a systemic disease, as Gb3 accumulation affects all cell types and tissues throughout the body. The principal phenotypic expression of cardiac involvement is LVH [37], and the resulting cardiomyopathy is one of the major determinants of prognosis [38]. Notably, FD is a “pan-cardiac disease,” since Gb3 accumulates in cardiomyocytes as well as conduction system cells, valvular fibroblasts, endothelial cells, and vascular smooth muscle cells [39]. Intriguingly, other mechanisms contribute to the development of LVH, far beyond the mere accumulation of Gb3, as sphingolipids account only for 1 to 2% of the total cardiac mass [40, 41]. Thus, it has been postulated that Gb3 accumulation might physically disturb the cardiomyocyte architecture and cause dysfunction, ultimately triggering intracellular signaling pathways leading to hypertrophy, apoptosis, necrosis, and fibrosis [42]. In this scenario, growing evidence suggests that inflammation has a key role in the disease progression.

Gb3 deposits, acting as antigens themselves, can activate invariant natural killer T cells leading to chronic inflammation and autoimmunity [43–46]. Inflammatory pathways are upregulated in different tissues and may be associated with apoptosis, impaired autophagy, and increase in pro-oxidative molecules, contributing synergistically to organ damage. Glycosphingolipids deposits may be recognized as antigens when presented to natural killer T (NKT) cells by CD1d-bearing antigen-presenting cells and may also behave as damage-associated molecular patterns (DAMPs) or cause DAMP production by injured cells. Therefore, Gb3 may be capable of activating Toll-like receptor (TLR)-4, the first line of innate host defense. In turn, TLR-4 activation triggers Notch1 signaling and the NF-κB pathway, all resulting in the production of pro-inflammatory cytokines [45–47].

### Evidence of systemic inflammation in Fabry disease

A role of chronic inflammation in FD is confirmed by evidence of increased expression of adhesion molecules in leukocytes and endothelial cells, such as soluble intercellular adhesion molecule-1, vascular cell adhesion molecule-1, P-selectin, plasminogen activator inhibitor [48], and CD31 in lymphocytes, monocytes, and granulocytes of Fabry patients as compared with healthy controls [49, 50]. Moreover, freshly isolated peripheral blood mononuclear cells and dendritic cells from FD patients showed increased expression of the proinflammatory cytokines IL-1β and TNF-α as compared to controls as well as a tendency to respond with higher levels of these molecules, including IL-6, upon LPS stimulation [46]. One of the consequences of inflammation is the generation of ROS, as seen in endothelial cells exposed to Gb3 in vitro. Gb3 itself, in a dose-dependent manner, induces oxidative stress, consistent with altered glutathione metabolism and high lipid peroxidation levels documented in FD [49–53]. Rozenfeld et al. also described a state of “leukocyte perturbation” characterized by a significantly higher percentage of lymphocytes and CD19 + cells and a reduced proportion of monocytes, CD8 + cells and myeloid dendritic cells in samples from Fabry patients compared with normal controls [50]. However, conflicting reports exists regarding the proportion of specific immune cell subpopulation [54] and further studies are needed.

The presence of a systemic chronic inflammatory state in FD is confirmed by the evidence of inflammation in organs typically affected by the disease, such as the kidney. In Fabry nephropathy, a relation between the cytokine synthesis profile and kidney fibrosis has been reported [55], as well as the role of TLR-4 and TGF-beta pathways triggered by Gb3. Moreover, although the mechanisms behind FD brain lesions is not completely understood, endothelial cell dysfunction and impaired vessel wall structure and function seem to be involved, and the role of inflammation in the latter phenomena is well established [43].

### Myocardial inflammation as a key feature of Fabry cardiomyopathy

**Fig. 3:**
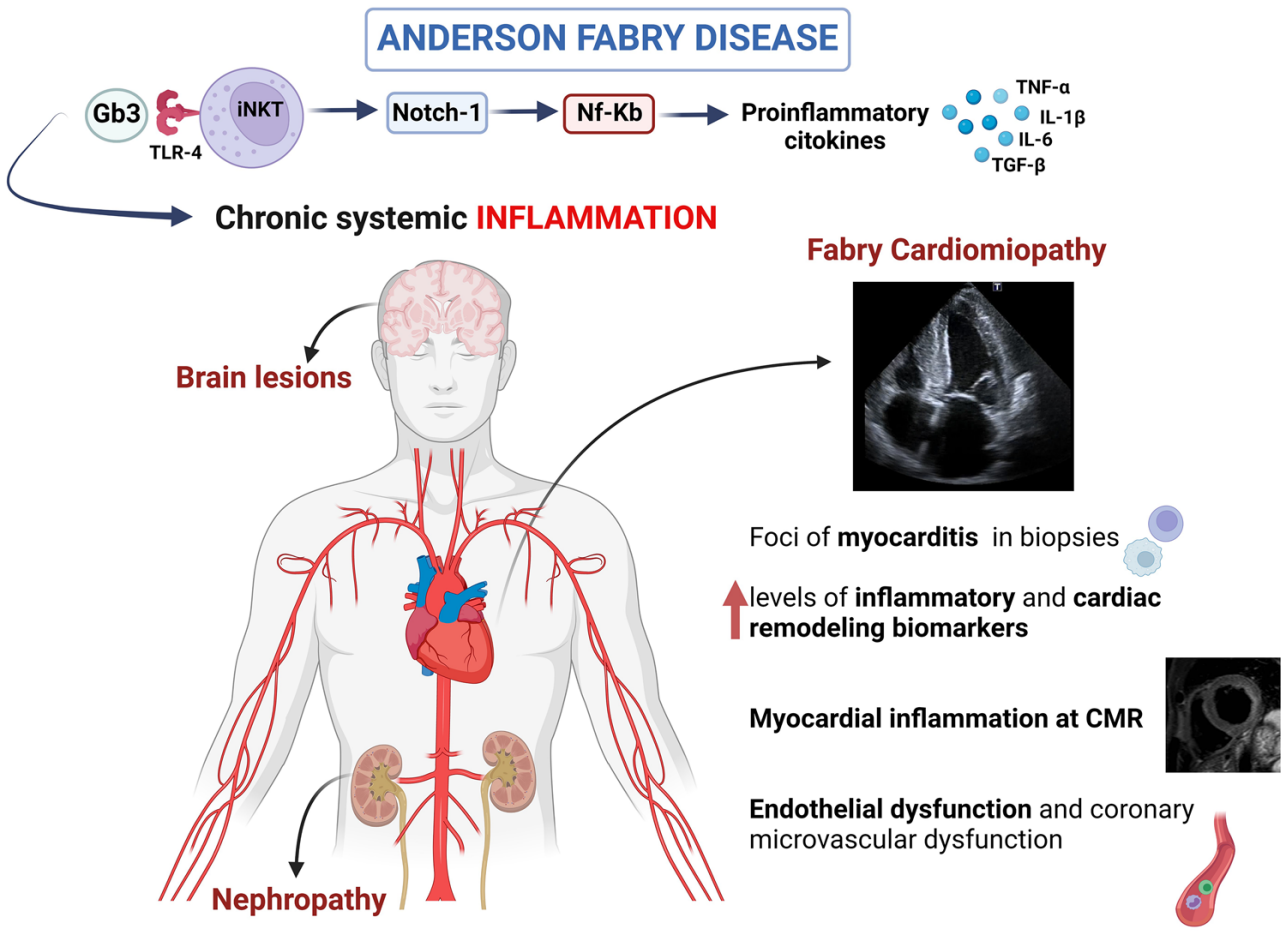
Role of inflammation in Anderson Fabry disease (FD). In FD Gb3 deposits act as antigens triggering a chronic inflammation state and autoimmunity in different tissues. In the heart, increased levels of inflammatory markers have been documented as well as sign of myocardial inflammation at cardiac magnetic resonance and foci of myocarditis in biopsies. Inflammation is involved in endothelial dysfunction and may trigger coronary microvascular dysfunction, a typical feature of Fabry cardiomyopathy (created with *BioRender*)

Increased levels of lymphocytes and macrophage-related markers CD68, CD163, and CD45 in endomyocardial biopsy samples from FD patients have been documented [55], supporting the novel concept of FD as an “inflammatory cardiomyopathy” [56] (Fig. 3). In a study on 78 subjects [56], endomyocardial biopsy specimens from FD patients revealed myocarditis (defined by CD3 + T lymphocytes > 7/mm2 associated with necrosis of glycolipid-laden myocardiocytes) in up to 56, and its presence was associated with angina, occurrence of arrhythmias, elevation of troponin I, and evidence of cell necrosis. Moreover, Yogasundaram et al. [8] demonstrated that inflammatory and cardiac remodeling biomarkers are elevated in FD and correlate with disease progression: the authors identify a “phenotype dominated by HFpEF with a key pathogenic role of systemic inflammation.” In this context, recent imaging techniques have been key in our understanding of the role of storage, inflammation, and fibrosis in different phases of the disease. Nordin et al. [57] performed a study combining blood and imaging biomarkers, showing that LGE T2 was very high in FD compared to HCM and chronic myocardial infarction, and higher than normal in every FD case. Moreover, troponin elevation only occurred when there was LGE. The strongest predictor of troponin release was T2 in the basal inferolateral wall, suggesting that LGE in that site was not a simple scar, but a focus of inflammation.

### Inflammation and vascular involvement in Fabry cardiomyopathy

Vascular involvement and endothelial dysfunction have been largely described in FD [58]. Storage of Gb3 within the media layer of the arteries promotes cell proliferation and fibrotic remodeling of the arterial wall, leading to increased stiffness and consequent shear stress. This may increase the expression of angiotensin 1 and 2 receptors in endothelial cells, ROS production, NF-κB, β-integrin, and cyclooxygenase 1 and 2 activity and decrease nitric oxide synthesis [59]. These mechanisms contribute to CMD, an important feature of Fabry cardiomyopathy. CMD has been described irrespectively of LVH and gender and may also represent the only sign of cardiac involvement [60], especially in females. The pathophysiological role of inflammation in CMD has been investigated in different clinical scenarios [61, 62], while the relation between the inflammatory profile and the presence and extent of CMD in Fabry cardiomyopathy has not been investigated so far. However, intriguingly, Knott et al. [63] demonstrated that at CMR with perfusion mapping areas of elevated T2 signal, i.e., areas of local inflammation, were the same of reduced myocardial blood flow, supporting the hypothesis of a “vicious circle” connecting inflammation, CMD, and myocardial injury.

### Impact of enzyme replacement therapy on inflammation

Unlike HCM, specific treatments are available for FD, including enzyme replacement therapy (ERT) and oral chaperon therapy for amenable mutations. ERT can slow disease progression and improve life expectancy, when started in a timely fashion [64]. However, whether it also modulates the immune system to limit inflammation is unresolved. Currently available studies on this topic are still conflicting, and comparisons between the effect of the two types of ERT (agalsidase alfa and beta) are limited by important methodological biases, including the heterogeneity of the enrolled populations [51–53].

Proteomic studies in animal models and humans aimed to assess the immunomodulatory effects of ERT: agalsidase beta normalizes the expression of genes associated with inflammation and vascular and renal function in Fabry mouse models [65]. Similarly, in humans, urinary proteome studies revealed a reduction in proinflammatory proteins, such as uromodulin and prostaglandins, in patients who treated with agalsidase beta [66]. However, in another gene expression study, immune and inflammatory pathways were found to be upregulated after agalsidase beta infusion [67]. Regarding the effect of ERT on the expression of pro-inflammatory cytokines, such as IL-1β, IL-6, and TNF-α, De Francesco et al. [46] observed no significant difference in treated vs untreated FD patients, whereas other authors have reported a significant reduction in serum levels of pro-inflammatory cytokines and oxidative stress markers following agalsidase alfa therapy [68]. In addition, no difference in leukocyte populations has been demonstrated between FD-untreated patients and those who received agalsidase alfa [50]. Thus, further studies are warranted to explore the impact of ERT on the immune system and inflammatory processes in FD. To date, the effects of chaperone therapy on inflammation are completely unknown.

## Cardiac amyloidosis

The amyloidoses are a group of diseases caused by misfolded proteins resistant to the body’s catabolic processes, which deposit extracellularly in different tissues leading to organ dysfunction [4]. More than 30 proteins that can form amyloid have been identified in humans. Amyloidosis may be caused by deposition of an intrinsically abnormal protein (e.g., hereditary transthyretin [hATTR] amyloidosis and acquired systemic immunoglobulin light-chain [AL] amyloidosis), prolonged exposure to excess of a normal protein (e.g., reactive systemic [AA] amyloidosis and β2-microglobulin dialysis-related amyloidosis), or by the ageing process (e.g., wild-type transthyretin amyloidosis [ATTRwt]). The most frequent type is AL amyloidosis, although recent studies suggest that ATTR may be more prevalent than previously thought in elderly people [69]. In AA amyloidosis [70], deposits are composed mainly of the serum amyloid A (SAA) protein, an apolipoprotein that serves as a dynamic acute phase reactant. It is synthesized by hepatocytes in response to various proinflammatory cytokines, such as TNF-α, IL-1 and IL-6. In this setting, inflammation is the main pathogenic mechanism but restrictive cardiomyopathy is extremely uncommon [71], probably due to the peculiar organ tropism of the protein. Conversely, heart involvement is the leading cause of morbidity and mortality in AL and TTR amyloidoses [72]. Cardiac amyloidosis typically presents as hypertrophic-restrictive cardiomyopathy leading to HF [4].

### AL amyloidosis

In AL amyloidosis, HF symptoms are often more severe than in TTR amyloidosis, despite lesser degrees of LVH [69]. This is likely be due to a stronger cardiotoxic effect of circulating free light chains, promoting a myocarditis-like process, while TTR-related amyloidosis is more akin to a true cardiomyopathy, with longer and less aggressive clinical course despite more impressive phenotypes [73]. In vitro studies showed that light-chain fibrillar aggregates can be cytotoxic and arrest the growth of an immortalized human cardiomyocyte cell line, called human RFP-AC16 cardiomyocytes [74]. Fibrils cause a “priming” immune response in adipose-derived mesenchymal stromal cells associated with interferon related genes, as shown by transcriptome analysis which have revealed an upregulation of innate immune-associated transcripts (chemokines, cytokines, and complement).

### TTR amyloidosis

Several studies support the hypothesis that TTR deposits trigger production of proinflammatory cytokines in hATTR patients (TNF-α, IL-1β, IL-8, IL-33, IFN-β, and IL-10) [75]. Moreover, studies on cardiac fibroblasts showed that TTR deposited in tissue extracellular matrix may affect the structure, function, and gene expression of these cells [76]. Fibroblasts cultured on deposited TTR showed disorganized cytoskeletal and nuclear structure as well as increased rates of proliferation and migration, while transcriptional sequencing and cytokine proteomic analysis revealed an upregulation of inflammatory genes, enhancing subsequent fibrosis. Azevedo et al. [75] found that *asymptomatic* patients with familial amyloid polyneuropathy (FAP) present high levels of IL-33, IL-1β, and IL-10, suggesting that inflammation has a role in the early stages of the disease. Suenaga et al. [77] confirmed the presence of a pro-inflammatory state in FAP hATTR patients and asymptomatic carriers as compared to healthy controls, with the former showing higher IL-6 levels. In addition, they also determined whether TTR deposits trigger production of pro-inflammatory cytokines ex vivo. They found that control-derived CD14 + monocytes and induced pluripotent stem cell–derived myeloid lineage cells from controls and FAP patients, dose-dependently produced IL-6 under mutated and aggregated TTR conditions. However, data on IL-6 are contradictory, as in another study its levels were comparable in hATTR FAP patients and asymptomatic gene carriers compared to healthy controls [75]. Furthermore, in a cohort of patients with overt cardiomyopathy, Hein et al. [78] found elevated IL-6 levels in ATTRwt patients, but not in hATTR carriers or hATTR cardiomyopathy patients. The authors speculated that in patients with preferential cardiac involvement of TTR amyloidosis, IL-6 seems to be a marker of HF rather than have a causative role. In the same study, IL-6 levels were associated with cardiac outcome at univariate analysis but did not retain an independent value at multivariable analysis over established risk predictors.

### AL and TTR amyloidoses: comparative studies

The first and only study investigating *histological evidence* of myocardial inflammation and its prognostic role in TTR and AL amyloidosis was performed by Siegismund et al. [9] who found a high prevalence (48%) of intramyocardial inflammation. Notably, a higher mortality rate was observed in patients with evidence of inflammation at endomyocardial biopsy and with AL-type amyloidosis. When AL and ATTR patients were stratified based on biopsy results, the presence of inflammation did not affect prognosis in TTR amyloidosis, while specifically the combination of inflammation *and* AL amyloid was associated with distinctively more severe outcomes. Thus, the authors hypothesized that an additional immunosuppressive therapy, aimed at controlling the inflammatory process before immune-mediated myocyte injury occurs, may have a beneficial effect in patients with cardiac AL amyloidosis. Another histological study [79] showed evidence of cardiac inflammation in as much as 42% patients with amyloidosis, specifically in 27% of ATTR-amyloidosis, 70% of AL-lambda, and 28% of AL-kappa amyloidosis. A significant infiltration of CD3 + T cells, CD68 + macrophages, and enhanced expression of MHCII and ERK1/2 were documented, with the latter likely playing a role in the onset of apoptotic cardiomyocytes and myocardial damage.

Koteca et al. [80] investigated the presence of myocardial edema by means of CMR T2 mapping in patients with AL and TTR amyloidosis to determine its prognostic significance in the two subtypes. They found that myocardial T2 was increased in amyloidosis, with the highest values observed in untreated AL patients. Intriguingly, myocardial T2 was predictive of prognosis in AL amyloidosis even when adjusted for extracellular volume and NTproBNP, but not in ATTR. As suggested by the authors, these findings support the concept of AL amyloidosis not being a disease due to pure infiltration, but one in which additional mechanisms contribute to the high mortality rate.

## Summary and future perspectives

Inflammation plays a pivotal role in a broad spectrum of conditions that injure the heart muscle, and accumulating evidence reveals that in cardiomyopathies with hypertrophic phenotype, inflammatory pathways are upregulated.

In HCM, inflammation likely plays a role in the complex interplay between genotype and phenotype, favoring the development of myocardial fibrosis and affecting disease progression and prognosis. Histological and CMR studies have proven myocardial inflammation as a common finding in Fabry cardiomyopathy, and within the heart, Gb3 is able to trigger inflammatory pathways involved in myocardial hypertrophy, fibrosis, and coronary endothelial and microvascular dysfunction. In both TTR and AL amyloidoses, inflammatory pathways may be triggered by TTR deposits and cytotoxic light chain, and current evidence suggests that in the AL subtype, myocardial inflammation is a common feature, and it is an independent predictor of prognosis.

In conclusion, inflammation could be a “fil rouge” across the spectrum of hypertrophic cardiac phenotypes even if currently it is not clear *if* and *how* these different diseases can be connected in terms of inflammation. Given these premises, the deep knowledge of molecular and cellular mediators involved is of outmost importance. More studies looking at cardiomyopathies through “immunologic lens” are to be encouraged, since they may provide clues for the identification of a high-risk subset of patients as well as could be the substrate for target treatments in a perspective of personalized medicine.

## Data Availability

Not applicable.
